# Supplementary material for: Is the Intrasexual Competition in Male Red Deer Reflected in the Ratio of Stable Isotopes of Carbon and Nitrogen in Faeces?
Source: Animals (Basel). 2023 Jul 24;13(14):2397. doi: 10.3390/ani13142397 (PMC10375991; doi:10.3390/ani13142397)
Supplement: Supplementary file 1 [file animals-13-02397-s001.zip › animals-2473810-supplementary.pdf]

animals

Supplementary materials

Table S1. Mean ± SD, minimum and maximum values of age, antler length (AL, cm), body length (BL, in cm), thoracic perimeter (TP, in cm), and the dark ventral patch size (DVP, in cm) per type of population (HC vs LC) as covariables included in LMM1 and LMM2. Percentage of parasitized and non-parasitized individuals in both HC and LC populations as a factor of LMM1 and LMM2.

Variable	Age	AL	BL	TP	DVP	$\delta^{13}C$	$\delta^{15}N$	Parasitized	
	Mean ± S.D. (Minimum / Maximum values)							Yes	No
HC (N= 46*)	3.37 ± 1.68 (2/8)	57.7 ± 9.58 (36.8/78.5)	181 ± 10.8 (154/201)	119 ± 6.16 (100/130)	36.8 ± 25.4 (0/73)	−26.3 ± 1.57 (−28.8/-21.7)	0.511 ± 1.28 (−2.17/2.87)	71.42 %	28.57 %
LC (N = 32*)	2.97 ± 1.52 (2/9)	52.3 ± 11.60 (36.4/82.6)	175 ± 12.1 (154/200)	114 ± 8.18 (100/132)	28.3 ± 20.4 (5/67)	−26.1 ± 1.30 (−28.9/-23.7)	2.25 ± 1.40 (−0.49/4.76)	80 %	20 %

* Faecal samples from 42 individuals of HC and 25 individuals from LC populations
